# Altered Spatial and Temporal Brain Connectivity in the Salience Network of Sensorineural Hearing Loss and Tinnitus

**DOI:** 10.3389/fnins.2019.00246

**Published:** 2019-03-19

**Authors:** Xiao-Min Xu, Yun Jiao, Tian-Yu Tang, Chun-Qiang Lu, Jian Zhang, Richard Salvi, Gao-Jun Teng

**Affiliations:** ^1^Jiangsu Key Laboratory of Molecular and Functional Imaging, Department of Radiology, Zhongda Hospital, Medical School of Southeast University, Nanjing, China; ^2^Center for Hearing and Deafness, University at Buffalo, Buffalo, NY, United States

**Keywords:** sensorineural hearing loss, tinnitus, functional connectivity, effective connectivity, salience network, non-auditory symptom

## Abstract

Sensorineural hearing loss (SNHL), sometimes accompanied with tinnitus, is associated with dysfunctions within and outside the classical auditory pathway. The salience network, which is anchored in bilateral anterior insula and dorsal anterior cingulate cortex, has been implicated in sensory integration. Partial auditory deprivation could alter the characteristics of the salience network and other related brain areas, thereby contributing to hearing impairments-induced neuropsychiatric symptoms. To test this hypothesis, we performed fMRI scanning and neuropsychological tests on 32 subjects with long-term bilateral hearing impairment and 30 well-matched Controls. Non-directional functional connectivity and directional Granger causality analysis were used to identify aberrant spatial and temporal patterns of connections targeting bilateral anterior insula and dorsal anterior cingulate cortex. We found that the left anterior insula showed decreased connectivity with right precentral gyrus and superior frontal gyrus. The connections between the dorsal anterior cingulate cortex and middle frontal gyrus, superior parietal gyrus and supplementary motor area (SMA) were also reduced. Relative to Controls, SNHL patients showed abnormal effective connectivity of the salience network, including inferior temporal gyrus, cerebellum lobule VI, lobule VIII, precentral gyrus, middle frontal gyrus and SMA. Furthermore, correlation analysis demonstrated that some of these atypical connectivity measures were correlated with performance of neuropsychiatric tests. These findings suggest that the inefficient modulation of the salience network might contribute to the neural basis of SNHL and tinnitus, as well as associated cognition and emotion deficits.

## Introduction

Sensorineural hearing loss (SNHL), which accounts for about 90% of all cases of hearing loss, results largely from the degeneration of cochlear sensory hair cells and their afferent auditory neurons (Charizopoulou et al., 2011; Li et al., 2017). SNHL is often accompanied by tinnitus (ringing in the ear), hyperacusis (loudness intolerance), impaired speech perception and poor temporal processing (Manohar et al., 2017; Paul et al., 2017). Since the sensory hair cells and auditory nerve fibers do not regenerate, the auditory deficits associated with SNHL are largely irreversible and can only be partially ameliorated with hearing aids or cochlear implants (Gordon et al., 2013). The auditory cortex, like other sensory cortices, forms parallel connections with other brain networks each with its own unique function(s) (Menon, 2011; Sepulcre, 2014). There is growing awareness that the lack of sensory input from the peripheral auditory system can disrupt neural circuits within and outside the classically defined auditory pathway, disruptions that could affect higher order cognitive, emotional, motivational, and attentional processes.

The salience network (SN), with major hubs in the dorsal anterior cingulate cortex (dACC) and anterior insula (AI), connects with subcortical and limbic structures (Seeley et al., 2007). It is well positioned to integrate internal and external stimuli, including those from the auditory pathway (Heywood et al., 2017). In patients experiencing auditory hallucinations, activity within the SN was perturbed and increased activation was present in the AI, suggesting greater relevance and attention to the internally generated auditory percepts (Sommer et al., 2008; Lefebvre et al., 2016). The AI is also activated in tasks designed to detect temporal disparities between auditory and visual stimuli (Bushara et al., 2001). Activity in the AI and dACC increased as auditory/visual information became more ambiguous and the task more difficult, suggesting that these structures might be of importance in sensory integration and cognition (Lamichhane et al., 2016). As the integrity of the SN was the basis of normal network functioning, white-matter damage in the SN could predict worse default mode network (DMN) function (Bonnelle et al., 2012; Luo et al., 2014; Sidlauskaite et al., 2016; Wu et al., 2016; Chang et al., 2017). As SNHL makes auditory processing more difficult, it deprives the SN of important auditory information needed for sensory integration, directing attention, decision making, and performing motivationally relevant tasks. Therefore, it should come as no surprise to learn that SNHL is associated with cognitive impairment, anxiety, depression and impaired executive function (Husain et al., 2011; Kronenberger et al., 2013; Basner et al., 2014). However, the potential mechanisms underlying SNHL-related neuropsychiatric symptoms and the relationships with the SN need to be further explored.

Functional connectivity (FC) is used to characterize the statistical dependence between the functional activity in two or more brain regions without making predictions about the causal nature of the interactions (Leavitt et al., 2012; Seth et al., 2015; Chen et al., 2017). By examining the time-lagged in the relationships between two or more brain regions, effective connectivity (EC) revealed by Granger causality analysis (GCA) tries to predict the direction of information flow in the network. These two powerful techniques can be used to identify the magnitude and direction of functional connections between different brain regions in a variety of neurological and psychiatric disorders (Demirci et al., 2009; Anderson et al., 2011; De Pisapia et al., 2012; Feng et al., 2016; Jiang et al., 2018). In the current study, we employed both FC and GCA to determine if SNHL disrupted non-directional and directional connectivity patterns using three major nodes in the SN as regions of interest (ROIs). We hypothesized that SNHL would disrupt the normal flow of neural activity within the SN and other parallel brain regions in the central nervous system. To determine if the disturbances in the SN were associated with a particular phenotype, we correlated the changes in FC and EC with performance on a series of cognition, depression, and anxiety tests.

## Materials and Methods

### Subjects

The study was conducted with two groups of subjects (Table 1); one group of 32 subjects had long term bilateral SNHL and the second group of 30 subjects, which formed the Control group, had clinically normal hearing. The SNHL sample included 22 males and 10 females with a mean age of 54.5 years ± 9.3 (*SD*), a mean body mass index (BMI) of 23.8 ( ± 3.3 *SD*), and a mean education level of 11.0 years ( ± 3.0 *SD*). The etiologies of long term bilateral SNHL were: unknown (*n* = 28), ototoxic drugs (*n* = 2) and chronic middle ear infection (*n* = 2). The Control group consisted of 14 males and 16 females with a mean age of 53.6 years ( ± 8.0 *SD*), a mean BMI of 23.2 ( ± 2.5 *SD*) and mean education level of 11.8 years ( ± 3.6 *SD*). Subjects were recruited into the study through clinical referrals though the outpatient clinics in Zhongda Hospital in Nanjing or through newspaper advertisements. All above procedures were approved by the Ethics Committee of Zhongda Hospital, Southeast University in Nanjing, China (2016ZDSYLL031.0). Every participant signed an informed consent form prior to participating in the study.

**Table 1:** Demographic and clinical characteristics of long-term bilateral SNHL and Control groups. Mean data (±*SD*) and *p*-values (^∗^*p* < 0.05; ^∗∗∗^*p* < 0.001) shown for SNHL and Control group.

	SNHL group	Control group	
Characteristic	(*n* = 32)	(*n* = 30)	*p*-value
Age (years)	54.5 ± 9.3	53.6 ± 8.0	0.698
Gender (male:female)	22:10	14:16	0.122
BMI	23.8 ± 3.3	23.2 ± 2.5	0.397
Education level (years)	11.0 ± 3.0	11.8 ± 3.6	0.348
Hearing loss durations (years)	8.0 ± 8.9	–	–
Framewise displacement	0.217 ± 0.097	0.227 ± 0.148	0.187
MMSE	29.6 ± 0.8	29.8 ± 0.6	0.315
SDMT	34.3 ± 12.5	41.5 ± 10.1	0.015*
AVLT-5 min	6.0 ± 2.7	6.8 ± 2.0	0.189
AVLT-20 min	5.9 ± 2.8	6.8 ± 1.8	0.140
SAS	36.1 ± 7.4	30.5 ± 3.7	0.001***
HAMD	6.1 ± 4.0	3.9 ± 3.2	0.024*

*Mean data (±*SD*) and *p*-values (^∗^*p* < 0.05; ^∗∗∗^*p* < 0.001) shown for SNHL and Control group. SNHL, sensorineural hearing loss; BMI, body mass index; MMSE, mini-mental state examination; SDMT, symbol digit modalities test; AVLT, auditory verbal learning test; SAS, self-rating anxiety scale; HAMD, hamilton depression scale*.

Only right-handed subjects between the ages of 20–65 years were included in the study. All subjects first underwent audiological testing to determine if they met the criteria for normal hearing (≤20 dB HL, octave interval from 0.25 to 8 kHz) for the Control group. The criteria for inclusion in SNHL were: (1) hearing loss duration> 1 year, (2) post-lingual hearing loss, (3) hearing thresholds ≥30 dB HL in both ears. Exclusion criteria included: abnormal visual acuity, Meniere’s diseases, acoustic neuroma, pulsatile tinnitus, drug or alcohol addiction, severe heart diseases, pregnancy, breast feeding, or MRI contraindications. Additionally, people were removed from this study if they had a history of depression or other psychiatric illness, Alzheimer’s disease, stroke, or head trauma.

### Audiological Tests

**Figure 1:**
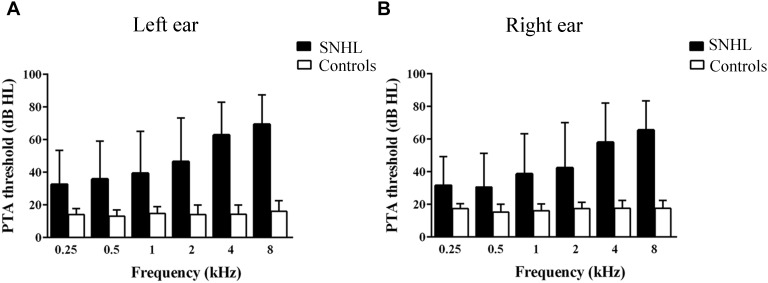
Hearing thresholds at 0.25, 0.5, 1, 2, 4, and 8 kHz. **(B)** Mean right-ear audiogram of SNHL group and Control group. **(A)** Mean left-ear audiogram of SNHL group and Control group.

A clinical audiologist performed pure tone audiometry (PTA) using a GSI-61 audiometer in the ENT department of Zhongda Hospital. Air conduction thresholds in the left and right ear were assessed at 0.25, 0.5, 1, 2, 4, and 8 kHz using standard clinical procedures (Figure 1). Impedance testing was conducted to rule out middle ear dysfunction test and conductive hearing loss.

### Neuropsychological Evaluation

Before MRI scanning, subjects underwent several cognitive and mental tests. The Mini-Mental State Examination (MMSE) is of high validation (Tombaugh and McIntyre, 1992) and can be easily performed to assess global cognition (Magni et al., 1996). Subjects with MMSE scores <26 were removed from our study. The Auditory Verbal Learning Test (AVLT) is a powerful tool for assessing episodic memory which consists of immediate and 20-min delayed recall, as well as recognition of 12 words (Brugnolo et al., 2014). It has been widely used for assessing cognition in dementia and pre-dementia conditions (Estevez-Gonzalez et al., 2003). The Symbol Digit Modalities Test (SDMT) was used to assess attention, processing speed and executive function (Benedict et al., 2017; Jaywant et al., 2018). The Self-Rating Anxiety Scale (SAS) contains 20 questions; 15 questions assess increasing anxiety levels and five questions assess decreasing anxiety levels (Yang et al., 2015). The Hamilton Depression Scale (HAMD) is considered as “gold standard” to capture core depressive symptoms in clinical research (Hamilton, 1967).

### MRI Scanning Protocol

MRI data were collected at the Radiology Department of Zhongda Hospital using a 3.0 T MRI system (Siemens, Erlangen, Germany) with an 8-channel receiver array head coil. Structural images were acquired with a T1-weighted 3D spoiled gradient-echo sequence: sections = 176, repetition time (TR) = 1900 ms, echo time (TE) = 2.48 ms, slices = 176, section thickness = 1.0 mm, flip angle (FA) = 90°, FOV = 256 × 256 mm^2^, acquisition matrix = 256 × 256. The fMRI data were obtained axially using a gradient-echo-planar (EPI) imaging sequence with the following parameters: 36 slices, TR/TE = 2000/25 ms, section thickness = 4.0 mm, FA = 90°, FOV = 240 × 240 mm^2^, acquisition matrix = 64 × 64. The fMRI data were acquired over a period of 8 min and 6 s and images were acquired in an interleaved order. Subjects wore earplugs and earphones to attenuate scanner noise (approximately 32 dB) and a head cushion was used to reduce head motion. Subjects were instructed to keep their eyes closed, remain awake, lie quietly, and avoid specific thoughts.

### Data Processing

Our data processing and analysis procedure have been described in detail in earlier publications (Cui et al., 2014, 2015). Briefly, functional data analyses were preprocessed using Data Processing Assistant for Resting-State software^1^ and SPM 12 toolbox^2^. The first 10 time points were discarded to allow for signal equilibrium and the remaining 230 consecutive volumes were used for subsequent analysis. Afterward, slice timing correction and realignment were performed. Structural and functional images were then coregistered to Montreal Neurological Institute (MNI) space, and data were resampled to 3 mm^3^. Six motion parameters, 6 temporal derivatives, 12 corresponding squared items were regressed using the Friston-24 parameter model to minimize the effect of head motion (Yan et al., 2013). The mean framewise displacement (FD) was computed from each time point for every subjects (Power et al., 2012; Van Dijk et al., 2012), and subjects with FD of >0.5 were excluded from the analysis. None of our subjects were removed because of excessive head motion and there was no significant difference in FD between SNHL and Control group (Table 1). Finally, data were smoothed with an isotropic Gaussian kernel (FWHM = 6.0 mm) and filtering from 0.01 to 0.08 Hz.

### Functional Connectivity

**Figure 2:**
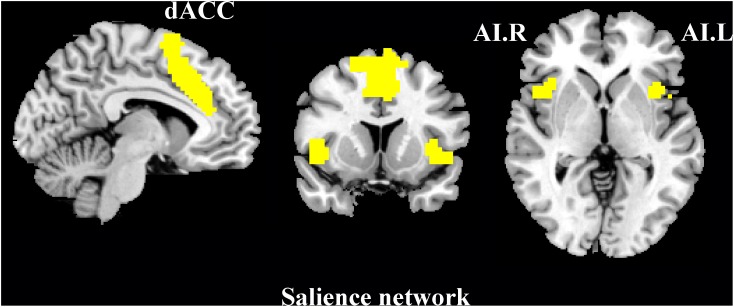
Schematic showing three ROIs, the dorsal anterior cingulate cortex (dACC), left anterior insula (AI), and right anterior insula (AI), as nodes in the salience network.

Functional connectivity was performed using REST software^3^. We used three primary nodes in the SN as ROIs (Figure 2) from the functional imaging in neuropsychiatric disorders lab at Stanford University^4^ (Shirer et al., 2012). The time-series of each region was averaged and correlated with every other voxel within the gray-matter mask. The FC correlation maps were converted using the Fisher r-to-z transform. Two-sample *t*-test was performed to explore the difference between SNHL patients and Controls. The statistical maps were thresholded at *p* < 0.001 at voxel level and *p* < 0.05 by Gaussian Random Fields (GRF) correction with age, gender and FD corrected. Significant clusters were mapped onto the surface of brain using BrainNet Viewer software^5^.

### Effective Connectivity

Effective connectivity was also analyzed by REST-GCA using the REST software and the same ROIs as described above. GCA is a statistical tool to depict the directionality flow of information in time series analysis (Stokes and Purdon, 2017). If the temporal progression of brain activity in one region allows the prediction of future time series activity in another brain area, it is assumed that the first brain region causally influences the second area (Granger, 1969). In present study, the time series from the left AI, right AI, and bilateral dACC were defined as the three seed time series, x, while the time series of all voxels in the brain were identified as the time series, y. Fx → y and Fy → x were calculated voxel by voxel as the linear direct influence from the time series x to y and from y to x, respectively. We transformed the residual-based F to a normally distributed F’, then converted the normally distributed *F*-values to standardized *Z*-scores (Z_x→y_ and Z_y→x_). Two Granger causality maps, (Z_x→y_ and Z_y→x_), were generated for each subject. Since we selected three seeds, six Granger causality maps were generated (left AI, Z_x→y_ and Z_y→x_, right AI with Z_x→y_ and Z_y→x_, and dorsal ACC, Z_x→y_ and Z_y→x_) for both the SNHL and Control groups. The GCA threshold was set at *p* < 0.01 at the voxel level and *p* < 0.05 corrected by GRF correction in two-sample *t*-tests with age, gender and FD corrected.

### Correlation Analysis

We also evaluated the relationships among the clinical variables and the functional MRI data. First, the clusters of significance in FC and GCA were extracted. Then, the mean *z*-values of the abnormal connectivity region masks (i.e., significant differences between the long term bilateral SNHL and Control groups) were calculated and used to perform a Person correlation analysis between the mean *z*-values of FC and each of the clinical variables (e.g., SDMT score, SAS scores, hearing ability, etc.,) using SPSS software (Version 18.0; Chicago, IL, United States). The significant *p*-value was set at *p* < 0.05, corrected for multiple comparison.

## Results

### Clinical Data and Neuropsychological Findings

Table 1 summarizes the demographic features, cognition and emotion status of the SNHL group and Control group. The two groups did not differ in terms of age, BMI, or education level. Consistent with our hypothesis, the SNHL group had significantly lower SDMT scores (*p* < 0.05), suggestive of poorer executive control or cognition among subjects with SNHL. The SAS scores (*p* < 0.001) and HAMD scores (*p* < 0.05) in the SNHL group were higher than those in the Control group suggesting greater anxiety and/or depression in subjects with long-term SNHL.

### Hearing Characteristic

All subjects in the control group had thresholds ≤20 dB HL at all frequencies in both ears (Yang et al., 2014) and normal middle ear function (Gwer et al., 2013); and subjects with SNHL had bilateral hearing thresholds ≥30 dB HL for more than 1 year (Figure 1A,B). Most SNHL patients had moderate to severe, sloping high frequency hearing loss although a few had profound hearing loss in one or more ears.

### Functional Connectivity

**Figure 3:**
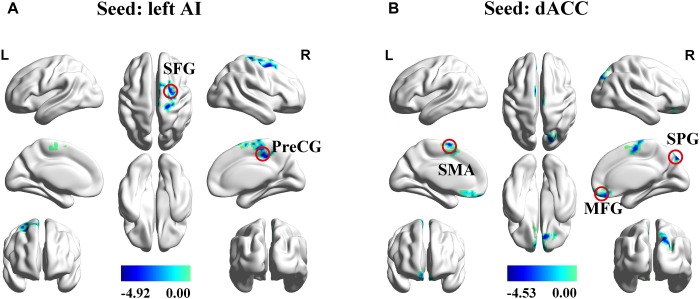
Significant differences in functional connectivity between SNHL and Control group with ROIs in the left AI, right AI, and dACC. **(A)** Reduced functional connectivity using the left AI as the seed. **(B)** Reduced functional connectivity using the dACC as the seed. Heat maps shows *z*-values; threshold set at *p* < 0.001 at the voxel level and *p* < 0.05 after Gaussian Random field correction. AI, anterior insula; dACC, dorsal anterior cingulate cortex; SFG, superior frontal gyrus; PreCG, precentral gyrus; SMA, supplementary motor area; MFG, superior frontal gyrus; SPG, superior parietal gyrus; L, left; R, right.

The left AI, right AI, and dACC were used as three nodes in our ROI analysis. We compared FC matrices in the SNHL and Control groups. SNHL did not lead to an increase FC between any of our three ROIs and other brain regions. Instead, we observed significant decreases in FC in the SNHL group compared to the Control group (Figure 3A,B and Table 2, *p* < 0.001, GRF corrected). The left AI showed decreased FC with the right precentral gyrus (PreCG) and superior frontal gyrus (SFG). Moreover, the dACC showed weakened connections with the right medial frontal gyrus (MFG) and superior parietal gyrus (SPG), as well as the left supplementary motor area (SMA).

**Table 2:** Regions of decreased functional connectivity in the SNHL group compared to the control group with the seed ROIs in the left AI, right AI, and dACC.

Seed			MNI coordinate	Peak	Cluster
region	Brain region	BA	x,y,z (mm)	*t*-score	size
Left AI	R precentral gyrus	6	18,-27,69	-4.4713	180
	R superior frontal gyrus	6	27,6,60	-4.9222	98
dACC	R medial frontal gyrus	11	12,39, -24	-4.0982	158
	R superior parietal gyrus	19	18, -84,45	-4.1949	154
	L supplementary motor area	6	-3,0,66	-4.5348	161

*BA, Brodmann area; MNI, Montreal Neurological Institute coordinates (MNI); AI, anterior insula; dACC, dorsal anterior cingulate cortex; L, left; R, right*.

### Effective Connectivity

**Figure 4:**
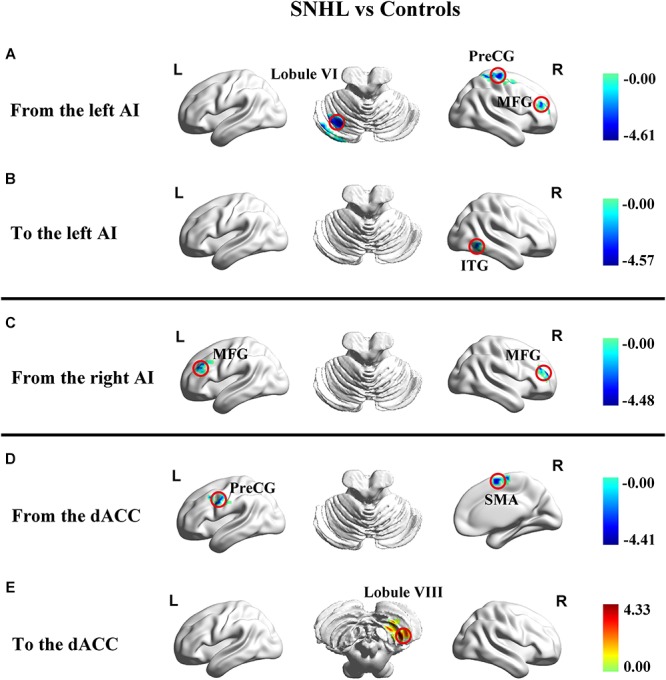
Changes in effective connectivity in the SNHL group compared to the control group using three key nodes in the salience network as ROIs (left AI, right AI, and dACC). **(A)** Decreased outflow effective connectivity from the left AI to left cerebellum lobule VI and right PreCG, MFG, and ITG highlighted by blue color; **(B)** Decreased inflow effective causal connectivity from right ITG to dACC highlighted by blue color; **(C)** Decreased outflow effective connectivity from the right AI to left and right MFG highlighted by blue color; **(D)** Decreased outflow effective connectivity from the dACC to the left PreCG and right SMA highlighted by blue color; **(E)** Increased inflow effective causal connectivity from the right cerebellum lobule VIII to the dACC highlighted by red color. Heat maps shows *z*-values; threshold set at *p* < 0.01 at the voxel level and *p* < 0.05 after Gaussian random field correction. SNHL, sensorineural hearing loss; AI, anterior insula; dACC, dorsal anterior cingulate cortex; PreCG, precentral gyrus; MFG, middle frontal gyrus; ITG, interior temporal gyrus; SMA, supplementary motor area.

In addition to assessing the magnitude of FC, GCA attempts to identify the directionality or presumed causality of information flow in a network (Goebel et al., 2003). We used GCA to determine if SNHL disrupted FC and its directionality in our three ROIs. Compared to Controls, the SNHL group demonstrated decreased outflow GCA values from the left AI to the left cerebellum lobule VI, right MFG and right PreCG (Figure 4A). The inflow from right inferior temporal gyrus (ITG) to the left AI was also decreased (Figure 4B). Meanwhile, the outflow from the right AI to bilateral MFG was reduced in SNHL subjects (Figure 4C). Significant changes in directional connectivity were also evaluated using the dACC as the ROI. We observed decreased outflow from the dACC to the left PreCG and right SMA, but increased inflow from right cerebellum lobule VIII (Figure 4D,E and Table 3).

**Table 3:** Changes in effective connectivity in the SNHL group compared to the control group using three key nodes in the salience network as ROIs (left AI, right AI, and dACC).

		MNI coordinate	Peak	Cluster
Brain region	BA	x,y,z (mm)	*t*-score	size
Casual outflow from left AI to the rest of brain (X to Y)				
L cerebellum lobule VI	19	-24, -69, -21	-4.6058	89
R middle frontal gyrus	45	45,42,18	-3.9075	140
R precentral gyrus	6	30, -21,63	-4.498	131
Casual inflow to the left AI from the rest of brain (Y to X)				
R inferior temporal gyrus	37	60, -51, -21	-4.569	80
Casual outflow from right AI to the rest of brain (X to Y)				
L middle frontal gyrus	46	-42,42,27	-3.8855	88
R middle frontal gyrus	46	33,51,18	-4.4832	141
Casual outflow from dACC to the rest of brain (X to Y)				
L precentral gyrus	6	-45,0,33	-4.2246	93
R supplementary motor area	6	3,0,60	-4.4094	97
Casual outflow to dACC from the rest of brain (Y to X)				
R cerebellum lobule VIII	-	30, -51, -51	4.3345	56

*BA, Brodmann area; MNI, Montreal Neurological Institute coordinates (MNI); AI, anterior insula; dACC, dorsal anterior cingulate cortex; L, left; R, right*.

### Correlation Analysis

**Figure 5:**
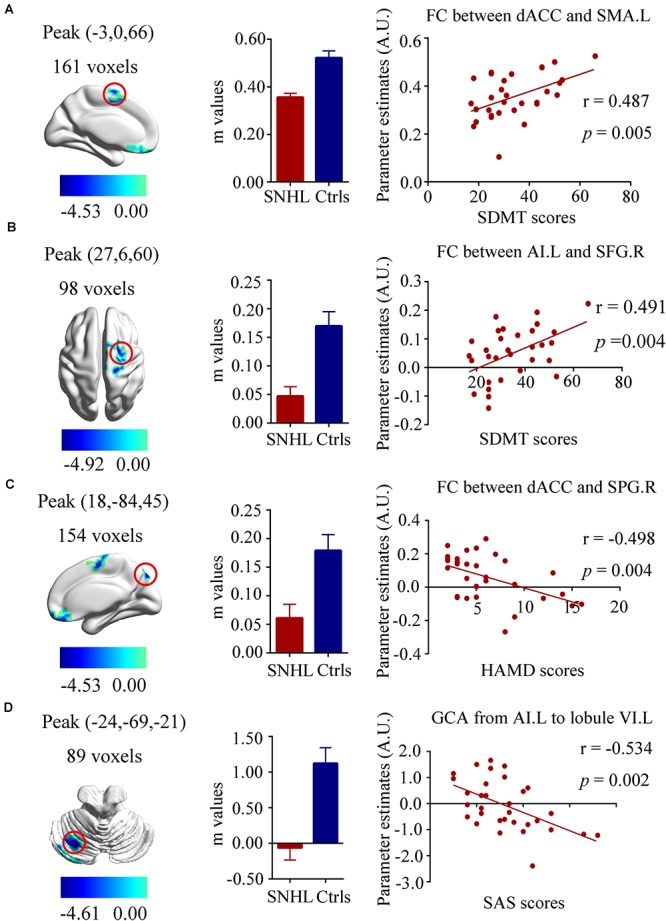
Relationships between fMRI and clinical neuropsychiatric scales. **(A)** Positive correlation between the SDMT scores and functional connectivity of dACC and left SMA (*r* = 0.487, *p* = 0.005); **(B)** Positive correlation between the SDMT scores and functional connectivity of left AI and right SFG (*r* = 0.491, *p* = 0.004); **(C)** Negative correlation between the HAMD scores and functional connectivity of left dACC and right SPG (*r* = –0.498, *p* = 0.004); **(D)** Negative correlation between the SAS scores and the decreased effective connectivity from the left AI to the left cerebellum lobule VI(*r* = –0.534, *p* = 0.002). SNHL, sensorineural hearing loss; Ctrls, controls; AI, anterior insula; dACC, dorsal anterior cingulate cortex; SMA, supplementary motor area; SFG, superior frontal gyrus; SPG, superior parietal gyrus; SDMT, symbol digit modalities test; HAMD, hamilton depression scale; SAS; self-anxiety scale.

Our neuropsychological data indicated that SNHL was associated with decreased executive function (SDMT scores), increased anxiety (SAS scores) and depression scores (HAMD scores) as noted in Table 1. To determine if these neuropsychological deficits were associated with the FC and EC changes, we correlated the SDMT, SAS, and HAMD scores with the changes in FC and EC for each of the three ROIs using voxel-wise methods. The SDMT performance showed positive correlations with dACC-left SMA FC (*r* = 0.487, *p* = 0.005, Figure 5A) and left AI-right SFG FC (*r* = 0.491, *p* = 0.004, Figure 5B). The connectivity between dACC and right SPG was negatively correlated with HAMD scores (*r* = -0.498, *p* = 0.004, Figure 5C). In addition, higher SAS scores were correlated with lower GCA values from left AI to left cerebellum lobule VI (*r* = -0.534, *p* = 0.002, Figure 5D).

## Discussion

To date, little research has focused on the spatial and temporal brain connectivity patterns in subjects with long-term bilateral SNHL. This is the first study combing non-directional FC and directional EC to uncover the abnormalities of intrinsic organization in brain regions using three key nodes of the SN as ROIs since the SN is found to play an crucial role in auditory processing (Li et al., 2015). Widely decreased connections were observed in the SNHL group. Significant alterations of EC appeared in multiple areas emanating from and to bilateral AI or dorsal ACC. In addition, some alterations in FC and EC were correlated with clinical measurements. Our results provide emerging evidence of changed functional architecture in the SNHL brain, providing new insight into the role of the SN and associated brain regions in deafness.

When processing speech, greater activation occurred in the AI as word recognition scores declined suggesting greater salience or attention in difficult listening situations (Harris et al., 2009). In order for effective aural communication to occur, a listener must selectively attend to the relevant acoustic signal in a noisy environment in order to from a coherent auditory stream, a task normal listeners perform effortlessly (Lee et al., 2008). The involvement of the SN has been implicated in tinnitus, as increased activity in the SN and enhanced insula-auditory cortex couplings might be associated with tinnitus percept and continuous awareness (Vanneste et al., 2013). Listeners with SNHL have great difficulty filtering out irrelevant acoustic information resulting in impaired auditory processing, diminished attention and an inability to use memory and cognition to fill in missing information in an auditory stream. Interestingly, we observed weakened non-directional and directional AI-PreCG connections in SNHL subjects. Although the PreCG is part of the posterior frontal lobe and traditionally functions as the primary motor cortex, evidence has indicated its role in auditory-associated events. Zhang et al. (2015) found decreased thalamic connectivity to the PreCG in tinnitus patients and the connections between the thalamus and insula have already been demonstrated in healthy adults (Namkung et al., 2017), so that decreased AI-PreCG connectivity in the SNHL group seems reasonable. However, there were some inconsistent findings, likely due to the various kinds of hearing impairments and disease durations. [^18^F]fluorodeoxyglucose (FDG)-PET, a quiet and reliable auditory imaging technique, demonstrated increased FDG uptake in the insula and PreCG within 72 h of onset of sudden SNHL. Glucose consumption in the PreCG was positively correlated with the speech discrimination scores (Micarelli et al., 2017). Compared to hearing non-dyslexics subjects, congenital deafness and dyslexic adults showed greater activation in the PreCG using a rhyme judgment task (MacSweeney et al., 2009). Taken together, these results suggest that the PreCG might participate in word recognition and phonological processing. Further experiments with these specific tasks in long-term SNHL could reveal the potential mechanism of the PreCG after partial hearing deprivation.

We found reduced AI-SFG FC and EC from AI to MFG in current study. Voxel-based morphometry (VBM) analysis (Husain et al., 2011) delineated gray matter changes in the SFG and MFG suggesting decreased use of executive control network (ECN) in chronic hearing loss patients, which supports our correlation between AI-SFG and SDMT scores (a symbol substitution test that examines an individual’s cognitive ability, including executive control and switching attention, which also requires oculomotor scanning and visuomotor coordination (Forn et al., 2009; Pascoe et al., 2018). Conversely, metabolic PET studies found increased FDG uptake in SFG and MFG in sudden hearing loss (Micarelli et al., 2017), indicative of enhanced neural activity in ECN regions. The increased metabolic activity might reflect enhanced conflict monitoring caused by the unexpected auditory input impairment and/or an emotional response in acute hearing loss period (Chen et al., 2013), while our aberrant ECN function was not immediate but occurred over time. Moreover, our connectivity results suggest abnormal interactions between the SN and the ECN (which includes dorsolateral prefrontal cortex) and casual flows from SN to ECN in SNHL. It has been reported that the SN plays a critical role in switching between the ECN and DMN, allocating information toward ECN and DMN (Sridharan et al., 2008). The SN has positive connectivity with the ECN and they can be co-activated during cognition tasks (Greicius et al., 2003). Here, hearing impairments altered inter-connectivity between SN and ECN, which could insights on cognitive control in SNHL.

It is important to note that the cerebellum is affected by SNHL. Emerging evidence shows that the cerebellum is not only associated with motor function, but also cognition processing and emotion control (Schmahmann and Caplan, 2006; Moreno-Rius, 2018). The cerebellum is considered to have a much border role in sensory and perceptual processing (Baumann et al., 2015). Except the primary auditory cortex, parts of the cerebellum are activated during various auditory tasks (Petacchi et al., 2005). Human and animal studies have also found activation in various regions of the cerebellum in its participation in hearing impairments, including tinnitus, hyperacusis and hearing loss (Stoodley and Schmahmann, 2009; Chen et al., 2015). Manganese-enhanced magnetic resonance imaging demonstrated increased spontaneous activity in portions of the cerebellum in rat with tinnitus, suggesting that it might serve as a tinnitus generator (Brozoski et al., 2007). Neuroimaging in unilateral hearing loss revealed enhanced and weakened connectivity between cerebellar networks and other systems (Zhang et al., 2018). In the present study, we observed weakened EC from left AI to left cerebellum lobule VI; this was negatively correlated with anxiety status revealed by SAS scores. Interestingly, lobule VI is considered part of the neural circuit of anxiety and fear (Lange et al., 2015).

EEG beta power of dACC was associated with a hyper-responsiveness state to non-noxious auditory stimuli in tinnitus and positively correlated with scores on tinnitus questionnaires (Song et al., 2014), which might contribute to presistent vigilance in tinnitus. In cochlear implant users, the dACC can be activated by sound stimuli and is engaged dual-stream auditory processing (Song et al., 2015), supporting its involvement in hearing impairments. The dACC showed decreased connections with the SPG and dACC-SPG FC was negatively correlated with HAMD scores. Contemporary views of brain function have emphasized the role of SPG in depression (Yang et al., 2017), tinnitus and hearing loss (Siedentopf et al., 2007), which correspond well to our correlation analysis. In our study, the outflow from dACC to PreCG was disrupted in subjects with SNHL. FC and independent component analysis (ICA) have suggested a robust relationship between the PreCG and dACC (Chen et al., 2018). White et al. (2010) found a weakened relationship between the PreCG and dACC in schizophrenia similar to our causal finding from the dACC and PreCG. We also observed the participation of SMA (falling with SFG) which is involved in motor action (Cona et al., 2017). Existing research shows that the dACC not only provides supplemental function to the SMA, but also resides at a higher level than the SMA (Hatanaka et al., 2003) in motor control, consistent with our correlation between dACC-SMA FC and SDMT performance. PET and fMRI studies found recruitment of the SMA in the perception of speech (Adank, 2012) and addressed the candidate role of SMA in auditory processing and auditory imagery (Lima et al., 2016). Moreover, the directed FC from the dACC to SMA is another aspect of a contribution from the frontal executive network (Asemi et al., 2015), and involvement of sensory process in executive control (Funahashi, 2001). Our data show that auditory deprivation results in abnormal directed and unidirectional connectivity from the dACC to SMA. However, further research is needed to elucidate the role of the SMA auditory processing pathway and disclose its role in SNHL-induced non-auditory symptoms.

### Limitation

There were several limitations in our preliminary study. First, this cross-sectional research does not provide direct evidence of causal relationships of functional MRI and hearing loss distress. Follow-up work using a longitudinal, within-subjects design are needed to confirm these findings. Second, the related approach should be expanded to sub-regions of bilateral AI and dACC, as well as other brain areas involved in ECN and emotional function. Third, further study using a larger sample size could be used to identify differences in the SN between SNHL subjects with tinnitus and without tinnitus. Finally, despite the fact that hearing protection was used by the subjects, scan noise from the MRI would influence spontaneous neural activity in the brain.

## Conclusion

To conclude, we identified disrupted functional and EC in the SN in long-term SNHL patients. In addition, cognitive impairments, depression and anxiety correlated with alterations in non-directional and directional connections. The imbalances between the SN and other brain regions could potentially disclose the neuropathological mechanism underlying hearing impairments and associated reorganization of complex networks.

## Data Availability

All datasets generated for this study are included in the manuscript and/or the supplementary files.

## Author Contributions

X-MX, collected the fMRI data, performed the analysis, and wrote the manuscript. JZ helped with data collecting. YJ and T-YT contributed to fMRI data analysis and discussion. C-QL helped with data processing. RS revised this manuscript. G-JT designed the MRI experiment and manuscript revision.

## Conflict of Interest Statement

The authors declare that the research was conducted in the absence of any commercial or financial relationships that could be construed as a potential conflict of interest.

## Footnotes

1 http://rfmri.org/DPARSF

2 http://www.fil.ion.ucl.ac.uk/spm

3 http://www.restfmri.net

4 http://findlab.stanford.edu/functional_ROIs.html

5 http://www.nitrc.org/projects/bnv/
